# Affective-Motivational Processes in TVET Internships: Challenge, Hindrance, School Support, and Vocational Persistence

**DOI:** 10.3390/bs16060995

**Published:** 2026-06-15

**Authors:** Cheng-Ze Hung, Stanley Y. B. Huang, Chien-Hsiang Huang

**Affiliations:** 1 Aircraft Engineering, Air Force Institute of Technology, Kaohsiung 820009, Taiwan; 2 Master Program of Financial Technology, Ming Chuan University, Taipei City 111013, Taiwan; 3 General Education Center, Chihlee University of Technology, New Taipei City 220305, Taiwan

**Keywords:** affective-motivational learning, educational well-being, work-integrated learning, curricular internship, challenge-hindrance demands, school support, technical and vocational education and training, vocational persistence

## Abstract

Curricular internships are affective-motivational learning contexts in which students encounter real workplace demands while educational institutions remain responsible for learning, engagement, and well-being. Responding to the Special Issue theme of emotion, motivation, and learning, this three-wave study used temporally separated self-report data to examine challenge demands, hindrance demands, and school support among 860 Taiwanese technical and vocational education and training (TVET) interns. Challenge demands were positively associated with work engagement, which was associated with innovative behavior. Hindrance demands were positively associated with burnout, which was associated with intention to seek work outside the trained vocational field. The hindrance demands-burnout association was weaker when school support was higher. The findings are compatible with treating school support as a curricular psychological resource that may help students interpret and manage obstructive internship conditions. More broadly, the study suggests that work-integrated learning systems may support vocational persistence by designing internships as supervised affective-motivational learning environments rather than as placements alone.

## 1. Introduction

Educational psychology increasingly treats emotion and motivation as constitutive elements of learning rather than as secondary outcomes. This perspective is especially important in work-integrated learning (WIL), where students move from classroom-based preparation into workplaces that can evoke enthusiasm, anxiety, effort, uncertainty, and occupational commitment at the same time. Curricular internships are therefore not merely administrative bridges between school and employment; they are emotionally charged learning environments in which students appraise whether workplace demands are meaningful, manageable, and connected to the occupational identity they are developing. These features directly connect the present study to the Special Issue theme because the educational value of an internship depends on how emotion, motivation, and learning are jointly shaped by the design and supervision of the placement.

From an affective-motivational design perspective, the same level of task difficulty can produce different learning consequences depending on whether students interpret the experience as a developmental challenge or as obstructive friction. A task that stretches skill use, problem solving, and responsibility may support engagement when feedback and role clarity are present. By contrast, unclear expectations, conflicting instructions, and poorly coordinated administrative requirements may consume psychological energy without helping students see progress toward competence. This distinction is central to educational psychology because it connects organizational conditions to learners’ emotional states, motivational investment, and persistence in the trained field.

Taiwan offers a particularly suitable context because higher technical and vocational education institutions are expected to maintain close linkages with industry and to cultivate practice-oriented capabilities (Ministry of Education, Taiwan, 2025). In many applied programs, an off-campus internship is not an optional enrichment activity but part of the formal route from study to work. This institutional position gives internships developmental importance. At the same time, the Taiwanese case speaks to a wider international issue: TVET and WIL systems in many countries ask schools, host organizations, and students to share responsibility for learning across institutional boundaries. A well-organized placement can help students read demanding work as evidence of growth in the field. A poorly organized placement can make the same transition feel confusing, unsupported, and misaligned with the occupation students are preparing to enter.

Prior Taiwan-based studies have linked internship experience to employability development, career self-efficacy, behavioral engagement in firm placements, internship satisfaction, and intention to remain in a sector (Chen et al., 2021; Hong et al., 2021; Liu, 2021; Wang, 2021). This literature indicates that internships matter for school-to-work transition, but it leaves a narrower educational-psychological question insufficiently specified: which internship demands support students’ affective-motivational involvement in learning, and which demands undermine well-being and vocational persistence while the placement is still underway? Addressing this question requires distinguishing between demands that are difficult because they are developmental and demands that are difficult because they obstruct learning.

This study draws on job demands-resources (JD-R) theory and the challenge-hindrance framework. Challenge demands require effort but can still be interpreted as meaningful, developmental, and connected to mastery. Hindrance demands also require effort, but they consume energy while making the learning value of work less clear. This distinction is especially relevant in internships because interns occupy a hybrid role: they are students, novice workers, and temporary members of host organizations at the same time. A busy placement is not necessarily a poor placement, but a busy placement that creates confusion without competence development can undermine the educational purpose of work-based learning.

This study contributes to educational psychology and WIL research in three ways. First, it separates developmental challenge from obstructive hindrance in curricular internships rather than treating placement intensity as a single dimension. Second, it tests motivational and well-being pathways in the same three-wave model, linking challenge demands to engagement and innovative behavior and hindrance demands to burnout and field-leaving intention. Third, it examines school support as a contextual educational resource that may weaken the harmful association between hindrance demands and burnout. These contributions are intended to be useful beyond Taiwan, because the same theoretical problem arises wherever workplace learning is embedded in formal curricula and schools remain accountable for learning quality after students enter host organizations.

## 2. Conceptual Background and Hypotheses

An internship does not become educational simply because it occurs outside the classroom. Learning depends on what the workplace affords, how students participate in practice, and how the school and host organization coordinate the experience (Billett, 2004). Boundary-crossing perspectives similarly suggest that students must translate expectations across school and workplace settings rather than simply transfer knowledge from one site to another (Akkerman & Bakker, 2011). Recent WIL scholarship emphasizes supervision, assessment, inclusion, and school-workplace coordination as conditions of placement quality rather than treating workplace exposure as sufficient by itself (Garant-Jones et al., 2024; Lasrado et al., 2024; Luk & Chan, 2024). In curricular internship systems, the placement is therefore both a labor-market encounter and a pedagogical arrangement. The student’s experience depends on the demands encountered at work and on the resources available for interpreting and managing those demands.

This framing also clarifies why school support is treated as a curricular psychological resource. In ordinary employment, workers may rely primarily on organizational supervisors and human resource systems. In a credit-bearing internship, however, students remain learners under the educational responsibility of the school. School support can therefore provide an interpretive and protective resource: it may help students decide whether difficulty is a normal part of learning, whether the placement is drifting away from educational aims, and whether help-seeking is legitimate. This logic provides the theoretical bridge between organizational demands and the affective-motivational states of learners.

Within the challenge-hindrance literature, challenge demands are effortful conditions that can be appraised as opportunities for mastery, growth, or meaningful contribution, whereas hindrance demands are more likely to be experienced as barriers to goal attainment (Cavanaugh et al., 2000; J. A. LePine et al., 2005; M. A. LePine, 2022). In educational terms, challenge demands can sustain motivation when they help students see a credible path from effort to competence. Hindrance demands can damage learning when students expend energy on ambiguity, conflicting expectations, or avoidable friction that provides little evidence of mastery. The distinction is useful because interns often need demanding work in order to learn. A placement that asks students to solve problems, coordinate with others, and apply skills under real constraints can support engagement if the work is understandable and feedback is available. By contrast, role ambiguity, conflicting requests, avoidable bureaucracy, and poorly coordinated expectations can drain effort without strengthening competence.

JD-R theory helps specify the expected processes. In the motivational pathway, developmental demands can foster engagement because effort is experienced as purposeful and connected to growth (Bakker & Demerouti, 2017; Demerouti et al., 2001). For interns, engagement should matter not only as positive involvement in work but also as a basis for proactive contribution. Engaged interns are more likely to invest discretionary energy, notice problems, generate ideas, and search for workable solutions. This expectation is consistent with evidence that experiential learning develops transferable skills through participation in social practice (Collins-Nelsen et al., 2022) and that workplace learning can support career exploration and initiative during an internship (Lu et al., 2024).

**H1.**  *Challenge demands are positively related to work engagement.*

**H2.**  *Work engagement is positively related to innovative behavior.*

**H3.**  *Work engagement mediates the positive relationship between challenge demands and innovative behavior.*

The well-being risk pathway concerns demands that obstruct learning. Hindrance demands are expected to increase burnout because students must spend energy coping with conditions that do not clearly contribute to development. In a curricular internship, this process may also affect vocational persistence. Students often use an internship as evidence about whether the trained field is a viable post-graduation destination. When the placement is marked by role conflict, unclear expectations, administrative friction, or poor task alignment, students may interpret the experience not as realistic preparation but as a warning about the field itself. Taiwan-based internship research has shown that internship satisfaction, employability development, career intention, and self-efficacy are linked to whether students can imagine a future in the sector (Chen et al., 2021; Liu, 2021; Wang, 2021).

**H4.**  *Hindrance demands are positively related to burnout.*

**H5.**  *Burnout is positively related to field-leaving intention.*

**H6.**  *Burnout mediates the positive relationship between hindrance demands and field-leaving intention.*

School support is treated as a contextual educational resource. In internships, schools do more than arrange placements. They communicate expectations, monitor student welfare, help students interpret workplace difficulties, and provide escalation routes when conditions become problematic. Support from the educational institution can help students distinguish demanding but developmental work from dysfunctional placement conditions. Work-placement research has also shown that support from supervisors and educational institutions matters for school-to-work transition, work readiness, and proactive career behavior (Okolie, 2022; Okolie et al., 2023). The present study extends this literature by testing whether school support moderates the affective cost of hindrance demands rather than only predicting positive career outcomes directly.

The buffering role of school support is especially relevant in Taiwanese TVET because internships often depend on cooperation among students, schools, and host organizations. Students may not have full employee status or the bargaining power of regular workers. They may also hesitate to challenge host practices when the internship is credit-bearing or linked to future employment opportunities. Visible and credible school support can reduce uncertainty, make help-seeking legitimate, and prevent students from interpreting preventable placement problems as personal failure. For that reason, school support is expected to weaken the positive association between hindrance demands and burnout.

**H7.**  *School support weakens the positive association between hindrance demands and burnout.*

**Figure 1:**
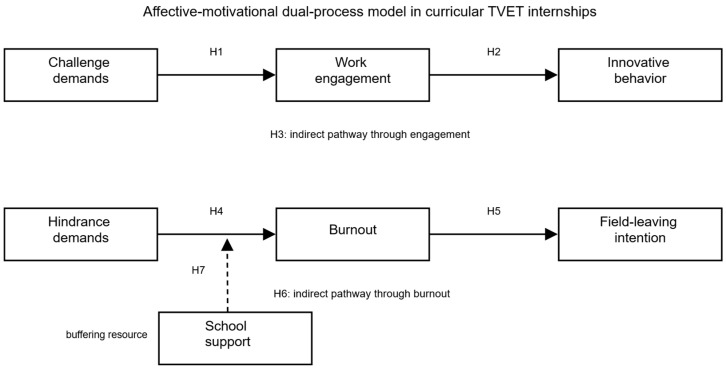
Hypothesized affective-motivational dual-process model of internship demands, school support, and vocational outcomes. Note. The figure presents the conceptual model only. Estimated standardized path coefficients are visualized in Figure 2.

The hypothesized dual-process model is summarized in Figure 1. To avoid conflating theory with results, Figure 1 presents only the conceptual paths; estimated standardized coefficients are reported later in Section 4.

**Figure 2:**
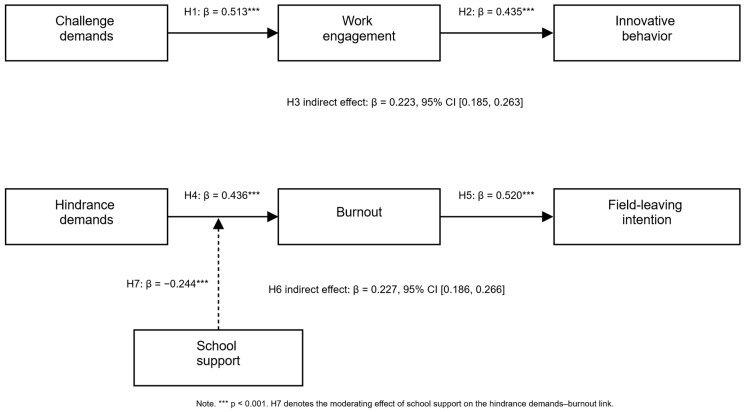
Estimated affective-motivational dual-process model. Note. Path coefficients are standardized estimates from the reported structural model; indirect effects are reported in Table 3. This figure is presented in Section 4 to distinguish empirical estimates from the hypothesized model in Figure 1.

## 3. Materials and Methods

### 3.1. Context and Participants

Participants were 860 students enrolled in placement-intensive higher TVET programs in Taiwan who completed curricular internships. The sampling frame consisted of students from five higher TVET institutions and 10 programs offering credit-bearing internships during 2025. Eligible participants were students who were currently undertaking or had just completed a formal curricular internship lasting 320 h and who were able to complete the three survey waves. Students were recruited through institutional internship offices and program coordinators. The initial Time 1 sample included 900 students, of whom 880 completed Time 2 and 868 completed Time 3. After responses were matched across waves and cases that could not support the three-wave model were excluded, the analytic sample comprised 860 matched responses, corresponding to 95.6% of the Time 1 sample. The dataset was de-identified before analysis.

The demographic and placement profile of the analytic sample was as follows. Of the participants, 45% identified as female and 55% identified as male. Participants’ mean age was 21.5 years (SD = 0.3); the narrow dispersion reflects the cohort-based structure of the internship period. Students were enrolled in business and management programs, and their internships were located in information-related industries. Because the de-identified analytic file did not retain per-case institution-level identifiers beyond the sampling frame, institution-by-institution distributions are not reported. These descriptors are reported to allow readers to assess the contextual specificity and transferability of the findings. The analytic sample should therefore be understood as a concentrated higher-TVET internship sample rather than a demographically broad national sample.

### 3.2. Procedure

Data were collected at three time points during the internship period through online survey links. Time 1 was administered in November 2025 and measured challenge demands, hindrance demands, and school support. Time 2 was administered in December 2025 and measured work engagement and burnout. Time 3 was administered in January 2026 and measured innovative behavior and field-leaving intention. Participants used anonymous matching information so that responses could be linked across waves without direct identifiers in the analytic file. Before participation, students received information about the research purpose, voluntary participation, confidentiality, and the use of de-identified data. This design separated predictors, mediators, and outcomes across measurement occasions, reducing single-occasion response bias while preserving confidentiality.

### 3.3. Measures

All items used a seven-point Likert response format. The questionnaire was administered in Chinese, with translation and back-translation used where adaptation was required. The adaptation process followed three steps: initial translation by a bilingual academic with education research expertise, independent back-translation by a second bilingual academic, and reconciliation by the research team to preserve conceptual rather than literal equivalence. Field-leaving intention was operationalized as intention to leave the trained vocational field rather than intention to leave a specific employer. This distinction is important because the study concerns vocational persistence after internship, not ordinary organizational turnover. Table 1 summarizes the constructs, item counts, example indicators, and source scales.

Challenge demands and hindrance demands were adapted from challenge-hindrance stressor research. Work engagement, burnout, and innovative behavior were adapted from established measures. School support was measured with internship-specific items focused on the educational institution’s responsiveness during placement. Because the construct refers to school-based support during credit-bearing workplace learning, the items were drafted after reviewing the internship-supervision literature and consulting internship supervisors. Content validity was examined by two university professors in an education-related department and two internship supervisors who evaluated item relevance, clarity, and fit with the internship context. The items were pilot tested with 20 TVET students, and wording was revised to reduce ambiguity before the main survey. In the analytic sample, the school support indicators showed acceptable reliability and convergent validity, with item loadings ranging from 0.769 to 0.818, Cronbach’s alpha of 0.809, composite reliability of 0.875, and AVE of 0.637. The measure of field-leaving intention was adapted from established turnover-intention items and reframed to refer to withdrawal from the vocational field for which students were being trained.

### 3.4. Analytical Strategy

Partial least squares structural equation modeling (PLS-SEM) was used to evaluate the measurement and structural models in SmartPLS 4.0. PLS-SEM was appropriate for the study because the model includes latent constructs, indirect paths, and an interaction term, and because the objective was a prediction-oriented explanation of internship outcomes rather than covariance-based global model-fit testing (Hair et al., 2022). Reliability and construct adequacy were assessed using Cronbach’s alpha, composite reliability, average variance extracted, and heterotrait–monotrait ratios (Fornell & Larcker, 1981; Hair et al., 2022; Henseler et al., 2015). The structural model tested two pathways: challenge demands →work engagement→innovative behavior and hindrance demands→burnout→field-leaving intention. The model also tested the moderating effect of school support on the association between hindrance demands and burnout. Indirect effects were evaluated with 3000 bootstrap resamples. The two supplementary direct paths reported in Section 4 were specified as a priori partial-mediation paths to avoid overstating full mediation. Common method concerns were addressed procedurally through temporal separation, anonymity, and the use of different measurement occasions. As a supplementary diagnostic, full-collinearity VIF values ranged from 1.101 to 1.642, below the commonly used 3.3 threshold for serious common-method concerns in PLS-SEM (Kock, 2015). This diagnostic does not eliminate the common-method limitation; it is reported alongside the study’s procedural remedies and the limitation that all focal measures were self-reported, and it is not treated as evidence of causal identification. Effect sizes were evaluated using Cohen’s f^2^ for the endogenous constructs. Because the design is observational, the results are interpreted as temporally separated associations rather than causal effects.

## 4. Results

Measurement quality was acceptable. Item loadings ranged from 0.746 to 0.887 in the retained measurement model. Cronbach’s alpha values ranged from 0.787 to 0.856, composite reliability values from 0.875 to 0.912, and average variance extracted values from 0.637 to 0.776. All heterotrait–monotrait ratios were below 0.85, with the highest value at 0.654. These results support internal consistency, convergent validity, and discriminant validity for the retained measures. Complete item-level loading evidence and the inter-construct HTMT matrix are reported in Supplementary Tables S1 and S2. Table 2 summarizes the measurement checks.

The lowest loading was 0.746, which remained above the conventional 0.70 guideline for established indicators. Accordingly, no item removal was required after the retained measurement model had been evaluated, and no substantive conclusion in the manuscript depends on a marginal indicator.

The structural results were consistent with the motivational pathway. Challenge demands were positively associated with work engagement (β = 0.513, t = 17.51, *p* < 0.001), and work engagement was positively associated with innovative behavior (β = 0.435, t = 12.73, *p* < 0.001). The indirect association linking challenge demands to innovative behavior through engagement was positive and statistically supported (β = 0.223, 95% CI [0.185, 0.263]). The corresponding effect sizes were f^2^ = 0.357 for challenge demands→work engagement and f^2^ = 0.189 for work engagement→innovative behavior; the supplementary direct path from challenge demands to innovative behavior was small (f^2^ = 0.016). These results indicate that demanding internship tasks were associated with stronger engagement when demands were consistent with development, and engagement was associated with innovative behavior.

The structural results were also consistent with the well-being risk pathway. Hindrance demands were positively associated with burnout (β = 0.436, t = 15.20, *p* < 0.001), and burnout was positively associated with field-leaving intention (β = 0.520, t = 17.73, *p* < 0.001). The indirect association linking hindrance demands to field-leaving intention through burnout was statistically supported (β = 0.227, 95% CI [0.186, 0.266]). The hindrance demands x school support interaction on burnout was negative (β = −0.244, t = −8.42, *p* < 0.001, 95% CI [−0.303, −0.187]), suggesting that school support attenuated the association between hindrance demands and burnout. Effect sizes for the burnout model were f^2^ = 0.270 for hindrance demands, f^2^ = 0.094 for school support, and f^2^ = 0.083 for the interaction term. Simple-slope probing showed that the hindrance demands-burnout association was stronger when school support was low (β = 0.680, t = 16.68, *p* < 0.001, 95% CI [0.600, 0.760]) and weaker, although still positive, when school support was high (β = 0.191, t = 4.69, *p* < 0.001, 95% CI [0.111, 0.272]).

**Figure 3:**
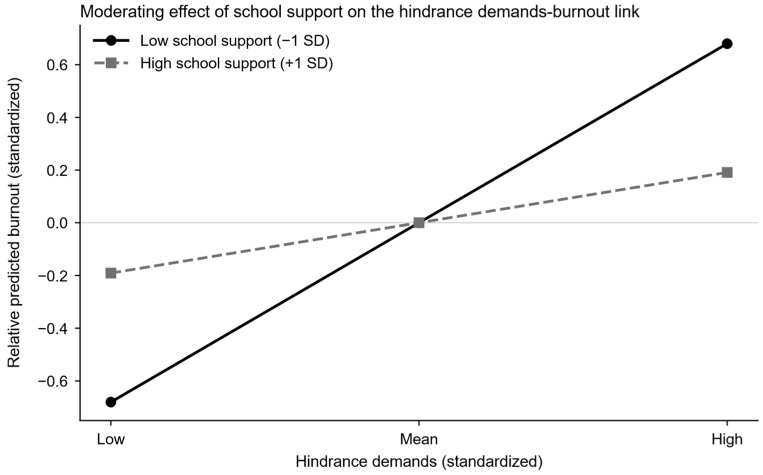
Graphical probing of the moderating effect of school support on the hindrance demands-burnout association. Note. The plot visualizes the standardized interaction by comparing the relative slope at low (−1 SD), mean, and high (+1 SD) school support.

Two supplementary direct paths were retained in the model as a priori partial-mediation paths. Challenge demands had a positive direct association with innovative behavior (β = 0.125, *p* < 0.001), and work engagement had a small negative association with field-leaving intention (β = −0.059, *p* = 0.040). These paths are reported to make the structural specification transparent and should not be read as replacing the hypothesized indirect mechanisms. In the field-leaving intention model, burnout showed a large effect size (f^2^ = 0.367), whereas the supplementary paths from work engagement (f^2^ = 0.005) and school support (f^2^ = 0.011) were small. The model explained 26.3% of the variance in engagement, 29.7% of the variance in burnout in the moderation model, 26.1% of the variance in innovative behavior, and 30.2% of the variance in field-leaving intention. Figure 2 and Figure 3 presents the estimated structural model and moderating effect of school support, and Table 3 reports the structural results.

The graphical probe indicates that hindrance demands were associated with higher burnout under both support conditions, but the increase was less pronounced when school support was high. This pattern is consistent with the interpretation of school support as a buffering curricular resource within the present observational design.

The pattern is meaningful in Taiwan because many higher TVET internships are curricular rather than optional. In that context, a weak placement is not simply a disappointing work episode; it can become part of how students judge the occupation for which they have been trained. At the same time, the mechanisms examined here are not unique to Taiwan. Any TVET or WIL system that places students in external organizations must manage the same pedagogical problem: schools remain responsible for learning quality even when day-to-day work is supervised elsewhere. The observed pattern therefore connects a behavioral model of demands and resources to an educational-psychological account of motivation, well-being, and persistence in a national training context while offering concepts that can be tested in other internship systems (Chen et al., 2021; Liu, 2021; Ministry of Education, Taiwan, 2025; Wang, 2021).

## 5. Discussion

This study examined internship quality as a question of affective-motivational educational design and supervision. The observed associations were consistent with, but do not by themselves establish, a dual-process interpretation. Challenge demands were associated with engagement and innovative behavior, whereas hindrance demands were associated with burnout and field-leaving intention. School support was associated with a weaker hindrance demands-burnout relationship. Because all hypotheses were statistically supported, the discussion focuses on theoretical boundary conditions rather than statistical confirmation alone: challenge may be useful when effort is connected to learning, hindrance may be harmful when effort is disconnected from competence development, and school support may matter most when students need help interpreting ambiguous workplace conditions. This pattern suggests why curricular internships should not be evaluated only by whether students are placed in workplaces. The more substantive question is how students experience, interpret, and regulate the demands of the placement.

The challenge pathway should be interpreted carefully. Challenge is not the same as overload. Students may benefit from demanding tasks when those tasks are connected to learning, feedback, and visible progress. Under those conditions, effort can become part of competence development, and engagement can become an affective-motivational signal that the placement is educationally meaningful. The acceptance of H1 to H3 therefore does not imply that programs should simply increase workload. Rather, it suggests that demanding tasks need to be framed as learning opportunities and supported by feedback, role clarity, and achievable progress markers. This interpretation is consistent with recent evidence that experiential learning develops transferable skills through participation in real-world settings (Collins-Nelsen et al., 2022) and that workplace learning can support career exploration during internships (Lu et al., 2024). For Taiwanese TVET programs, the implication is not to remove difficulty, but to make developmental demands understandable and supported.

The hindrance pathway points to a different problem. Hindrance demands did not resemble productive difficulty. They reflected friction that disrupted learning and well-being. Role ambiguity, conflicting requests, and avoidable administrative burden can make students spend effort without gaining a clearer sense of competence or field fit. The acceptance of H4 to H6 suggests that obstructive internship conditions are not merely inconveniences; they are associated with burnout and with weaker vocational persistence. In Taiwanese TVET, where internships are often part of formal preparation for employment, this pattern carries particular weight. If students experience the placement as confusing or poorly aligned with learning, burnout may become part of their vocational socialization. This extends previous Taiwan-based work linking internship satisfaction, employability development, and career self-efficacy to intention to remain in a sector (Chen et al., 2021; Liu, 2021; Wang, 2021).

School support was the most directly actionable resource examined in the model. Support was associated with a weaker hindrance demands-burnout relationship. The acceptance of H7 suggests that schools may reduce the psychological cost associated with obstructive placement conditions by helping students interpret problems, seek assistance, and distinguish normal developmental challenge from poor placement design. The finding is consistent with evidence that support during work placement matters for readiness for school-to-work transition and proactive career behavior (Okolie, 2022; Okolie et al., 2023). In practice, school support may include routine check-ins, role-clarity monitoring, responsive teacher contact, and clear procedures for escalation. Theoretically, the finding supports treating school support as more than a background administrative service: it is part of the affective-motivational architecture of WIL.

The study also refines how vocational persistence should be understood in internship research. Field-leaving intention does not refer to leaving a particular employer. It refers to the intention to seek work outside the trained vocational field. This distinction is important for internships because students are still forming occupational judgments. A negative internship does not necessarily determine an actual career exit, but it can influence whether the field appears credible and sustainable after graduation. The three-wave design strengthens temporal ordering, but the associations should not be read as proof that internship demands caused later vocational decisions.

A practical implication is that internship supervision should be proactive. Many placement problems accumulate through repeated small mismatches among task assignment, feedback, and role clarity. If schools wait until final reflection reports, engagement may already have declined, and burnout may have increased. Programs can schedule brief monitoring points during the early and middle phases of the internship. Students can be asked to identify one task that is stretching their competence and one condition that is obstructing learning. Host supervisors can be asked to explain the learning value of major tasks. School supervisors can then compare these accounts and intervene when expectations diverge.

This approach also helps supervisors distinguish challenge from hindrance in practice. A demanding task should not be treated as harmful simply because it is difficult. It becomes harmful when students cannot see its learning value, when expectations conflict, or when effort is spent coping with avoidable friction. Conversely, a task can be challenging and useful when effort is paired with feedback, role clarity, and a plausible link to professional growth. The distinction provides a practical vocabulary for site visits and student consultations.

For Taiwanese TVET institutions, this vocabulary is useful because school-industry partnerships often need to satisfy several purposes at once. Host organizations may expect interns to contribute to the daily workflow. Schools may expect evidence of learning outcomes. Students may expect occupational exploration and employability development. These purposes are compatible only when the placement is framed and monitored. Without such framing, students may be left to reconcile conflicting expectations alone. The present results suggest that this is not only an administrative inconvenience but also an affective-motivational risk, because unsupported hindrance demands were associated with burnout and burnout was associated with field-leaving intention. Internationally, the same logic applies to WIL systems that depend on distributed supervision across educational and workplace settings.

### Practical Implications

Three practical implications follow. First, programs can define what counts as a developmental challenge before placement begins. Students and host supervisors should know which tasks are expected to stretch competence and how progress will be supported. A short role-mapping checklist can help identify whether assigned tasks connect to program learning goals and whether students can see the learning value of effortful assignments. At the policy level, Taiwanese TVET authorities and institutions can use such checklists to move internship quality assurance beyond placement counts and toward evidence of supervised learning quality.

Second, schools can monitor hindrance demands during placement rather than only after placement. Brief check-ins can ask whether students understand what is expected, whether task assignments are consistent, whether feedback is available, and whether administrative requirements are interfering with learning. These questions are directly connected to the burnout pathway examined in the model.

Third, escalation channels can be made visible and credible. Students may hesitate to challenge host practices when the internship is credit-bearing or connected to future employment. A clear school contact can make help-seeking a normal part of internship learning rather than an exceptional complaint. This is particularly important when role ambiguity or conflicting requests arise.

Fourth, policy and institutional guidelines can treat school support as a required quality component of curricular internship rather than as an optional service. This recommendation is relevant outside Taiwan as well. In countries where WIL is expanding, institutions can adapt the present model to audit whether internships provide developmental challenge, minimize avoidable hindrance, and maintain credible channels for student support while students are off campus.

These recommendations do not require programs to remove difficulty from internships. They ask programs to separate productive challenge from avoidable hindrance and to make school support visible before problems become burnout. In many programs, such monitoring can be implemented by refining existing internship check-ins, supervision forms, and escalation procedures rather than by creating an entirely new administrative system. This underscores the practical value of applying an educational-psychological behavioral model to curricular internship design.

## 6. Limitations and Future Research

The study has several limitations. First, the three-wave design improves temporal ordering but does not establish causality. Second, all focal measures were self-reported. Although temporal separation reduces single-occasion response bias, common method inflation cannot be ruled out completely. Future research should incorporate supervisor ratings, placement records, or behavioral indicators of innovation and persistence. Third, although participant and placement descriptors were added, the de-identified analytic file did not retain more granular demographic, institutional, program-level, or host-organization identifiers. As a result, the study cannot examine whether the structural relations vary by gender subgroup, age subgroup, institution, program, or internship industry. The sample was also concentrated in business and management programs and information-related internship sites; therefore, generalization to other TVET fields should be made cautiously. Fourth, students may have been nested within programs or host organizations, but multilevel modeling was not feasible because program-level and host-organization identifiers were not available in the analytic file. Future studies should examine how specific school support practices operate through different mechanisms and for which student subgroups they are most effective. Fifth, because the study was conducted in Taiwan, cross-cultural replication is needed to examine whether similar affective-motivational processes appear in other TVET and WIL systems.

## 7. Conclusions

This study suggests that internship quality is partly a matter of affective-motivational supervision design. In Taiwanese TVET, challenge demands were associated with engagement and innovative behavior, whereas hindrance demands were associated with burnout and field-leaving intention. Higher school support was associated with a weaker hindrance demands–burnout relationship. The findings are compatible with the interpretation that internships may be most educational when schools remain actively involved in how workplace learning is framed, monitored, and supported. When that involvement is weak, routine friction may be associated with early intention to leave the training field rather than becoming a manageable part of learning at work. These findings are therefore relevant not only to Taiwan but also to international TVET and WIL systems that seek to combine workplace exposure with educational responsibility.

## Figures and Tables

**Table 1:** Measures used in the analysis.

Construct	Items	Example Indicator	Source/Note
Challenge demands	4	My internship tasks require me to use a high level of skill and knowledge.	Adapted from challenge–hindrance stressor work (Cavanaugh et al., 2000; J. A. LePine et al., 2005).
Hindrance demands	4	It is often unclear what is expected of me at the internship.	Adapted from challenge–hindrance stressor work (Cavanaugh et al., 2000; J. A. LePine et al., 2005).
School support	4	The school provides help when I face difficulties at the internship.	Internship-specific items capturing perceived backing from the educational institution.
Work engagement	3	At my internship, I feel bursting with energy.	Adapted from Schaufeli et al. (2006).
Burnout	3	After my work, I usually feel worn out and weary.	Adapted from Demerouti and Bakker (2008).
Innovative behavior	3	I generate creative ideas for difficult problems at work.	Adapted from Scott and Bruce (1994).
Field-leaving intention	3	I plan to look for a job in a different industry after graduation.	Adapted from established turnover-intention items (Allen et al., 2003) for field withdrawal.

Note. All items used seven-point Likert response options (1 = strongly disagree to 7 = strongly agree).

**Table 2:** Measurement quality and discriminant validity summary.

Indicator	Reported Value	Interpretation
Item loadings	0.746–0.887	All retained indicators met conventional loading guidelines.
Cronbach’s alpha	0.787–0.856	Internal consistency was acceptable across constructs.
Composite reliability	0.875–0.912	Composite reliability met common adequacy thresholds.
Average variance extracted	0.637–0.776	All AVE values exceeded 0.50.
Highest HTMT ratio	0.654	Discriminant validity was supported because all HTMT values were below 0.85.

**Table 3:** Structural results for the matched three-wave sample.

Path Test	β	t	95% CI	*p*	Decision
Challenge demands→Work engagement (H1)	0.513	17.51	—	<0.001	Supported
Work engagement→Innovative behavior (H2)	0.435	12.73	—	<0.001	Supported
Challenge demands→Work engagement→Innovative behavior (H3)	0.223	—	[0.185, 0.263]	—	Supported
Hindrance demands→Burnout (H4)	0.436	15.20	—	<0.001	Supported
Burnout→Field-leaving intention (H5)	0.520	17.73	—	<0.001	Supported
Hindrance demands→Burnout→Field-leaving intention (H6)	0.227	—	[0.186, 0.266]	—	Supported
Hindrance demands × School support→Burnout (H7, interaction)	−0.244	−8.42	[−0.303, −0.187]	<0.001	Supported
Challenge demands→Innovative behavior (supplementary direct path)	0.125	—	—	<0.001	Retained
Work engagement→Field-leaving intention (supplementary direct path)	−0.059	—	—	0.040	Retained

Note. Indirect effects were evaluated with 3000 bootstrap resamples. For indirect effects, statistical support was evaluated using 95% bootstrap confidence intervals; therefore, the table reports confidence intervals rather than t-values for those mediation tests.

## Data Availability

The data presented in this study are available on reasonable request from the corresponding author, subject to institutional privacy restrictions.

## Supplementary Materials

The following supporting information can be downloaded at: https://www.mdpi.com/article/10.3390/bs16060995/s1. Table S1: Item-level loading evidence; Table S2: Inter-construct HTMT matrix; Table S3: Full-collinearity VIF and f^2^ effect-size checks; and Table S4: Simple-slope probing of the moderation effect.
