# Supplementary material for: Affective-Motivational Processes in TVET Internships: Challenge, Hindrance, School Support, and Vocational Persistence
Source: Behav Sci (Basel). 2026 Jun 15;16(6):995. doi: 10.3390/bs16060995 (PMC13295619; doi:10.3390/bs16060995)
Supplement: Supplementary file 1 [file behavsci-16-00995-s001.zip › behavsci-4334543-supplementary.pdf]

Supplementary File S1. Measurement and Robustness Checks

The following tables report additional measurement and robustness evidence prepared from the de-identified analytic dataset used for the revised manuscript. The tables are intended to make the measurement and moderation evidence auditable without adding excessive detail to the main text.

Table S1. Item-Level Loading Evidence

Construct	Items	Loadings	Cronbach's alpha	Composite reliability	AVE
Challenge demands	CD1-CD4	.830, .746, .821, .810	.815	.878	.644
Hindrance demands	HD1-HD4	.817, .813, .797, .799	.820	.882	.651
School support	SS1-SS4	.818, .808, .769, .797	.809	.875	.637
Work engagement	WE1-WE3	.827, .872, .834	.798	.882	.713
Burnout	BO1-BO3	.836, .814, .863	.787	.876	.702
Innovative behavior	IB1-IB3	.864, .817, .832	.787	.876	.702
Field-leaving intention	TI1-TI3	.887, .873, .883	.856	.912	.776

Note. CD = challenge demands; HD = hindrance demands; SS = school support; WE = work engagement; BO = burnout; IB = innovative behavior; TI = field-leaving intention. The main manuscript reports the retained PLS-SEM measurement-model summary; this supplementary table provides item-level evidence for auditability.

Table S2. Inter-Construct HTMT Matrix

Construct	CD	HD	SS	WE	BO	IB	TI
CD	-						
HD	.204	-					
SS	.046	.075	-				
WE	.636	.105	.081	-			
BO	.060	.530	.279	.060	-		
IB	.434	.046	.071	.631	.047	-	
TI	.061	.352	.254	.050	.654	.056	-

Note. All HTMT values were below .85. The highest HTMT value was .654 for burnout and field-leaving intention.

Table S3. Full-Collinearity VIF and f^2 Effect-Size Checks

Check	Value	Interpretation	Use in manuscript
Full-collinearity VIF range	1.101-1.642	Below 3.3	Common method concern check
CD -> WE	$f^2 = .357$	Large	Motivational pathway
WE -> IB	$f^2 = .189$	Medium	Motivational pathway
CD -> IB supplementary	$f^2 = .016$	Small	A priori partial-mediation path
HD -> BO	$f^2 = .270$	Medium-to-large	Risk pathway
SS -> BO	$f^2 = .094$	Small-to-medium	Resource effect
HD x SS -> BO	$f^2 = .083$	Small-to-medium	Moderation
BO -> TI	$f^2 = .367$	Large	Risk pathway
WE -> TI supplementary	$f^2 = .005$	Very small	A priori partial-mediation path
SS -> TI supplementary	$f^2 = .011$	Small	Resource-related direct association

Note. Full-collinearity VIF was used as a statistical check for serious common method bias concerns. Effect-size interpretations are descriptive and should be read together with the theoretical model.

Table S4. Simple-Slope Probing for the Moderation Effect

School support level	Slope of HD -> BO	t	p	95% CI
Low school support (-1 SD)	.680	16.68	< .001	[.600, .760]
Mean school support	.436	15.20	< .001	[.380, .492]
High school support (+1 SD)	.191	4.69	< .001	[.111, .272]

Note. HD = hindrance demands; BO = burnout. The slopes show that the hindrance demands-burnout association remained positive but was weaker when school support was high.
